# ZBTB7A regulates CD95-mediated cell growth in colorectal cancer cell lines

**DOI:** 10.1371/journal.pone.0329958

**Published:** 2025-09-15

**Authors:** Yanhong Bie, Meiying Bie, Wang Liu, Shufang Tao, Hongyu Xing, Linlin Kuang, Yi Song

**Affiliations:** 1 Shanghai Jiading District Anting Hospital, Shanghai, China; 2 Shanghai Tianchen Rehabilitation Hospital, Shanghai, China; 3 Shanghai Baoshan Hospital of Integrated Traditional Chinese and Western Medicine, Shanghai, China; 4 Shanghai Institute of Immunity and Infection, Chinese Academy of Sciences, Shanghai, China; 5 Shanghai Jiading Maternal And Child Health Care Hospital, Shanghai, China; University of New Hampshire, UNITED STATES OF AMERICA

## Abstract

Zinc Finger and BTB Domain-containing7A (ZBTB7A) is a member of the transcription factor family that regulates the expression of numerous genes involved in cell proliferation and differentiation. Ubiquitously expressed in colon, stomach, and other tissues, ZBTB7A performs diverse functions including hematopoiesis, metabolism, and oncogenesis. Here we showed that ZBTB7A knockout inhibited the growth of colon cancer cells and reduced CD95 protein expression by decreasing CD95 mRNA transcription. Overexpression of ZBTB7A could partially restore both CD95 expression and CD95-mediated downstream Caspase2 and JNK2 expression. Furthermore, treatment with ZBTB7A specific inhibitor Curcumin effectively induced colorectal cancer cell death while reducing CD95 expression. These data indicate that ZBTB7A promotes colon cancer cell growth and survival, suggesting its potential as a therapeutic target for colorectal cancer.

## Introduction

Colorectal cancer is one of the most prevalent cancers worldwide [1], and the current treatment methods mainly include surgical resection, radiotherapy, and chemotherapy. However, approximately 20% of patients lose the opportunity for curative surgery due to late-stage detection [2]. As a result, molecular-targeted therapies have become increasingly critical for these patients in recent years.

ZBTB7A, also known as LRF, FBI1, FBI-1, TIP21, ZBTB7, MNDLFH, ZNF857A, pokemon, is a pleiotropic transcription factor that belongs to the POZ/BTB and Krüppel (POK) family, and it is associated with the occurrence and metastasis of various cancers and is widely expressed in colorectal cancer cells [3]. Previous study has reported that low ZBTB7A expression in colon and lung cancer patients is associated with decreased survival rates [3], and ZBTB7A also inhibits the activity of the tumor suppressor protein p53 in colorectal cancer cells [4], implying that ZBTB7A may regulate cell proliferation and death [5]. Thus, we aim to further investigate the role of ZBTB7A in colorectal cancer.

CD95 (also called Fas and APO-1) is a typical death receptor inducing cell apoptosis [6], but it has also been reported that CD95 could promote the growth of some tumors [7,8].Our screening has found that both CD95 and ZBTB7A are highly expressed in HCT116 colorectal cancer cells. Therefore, we are interested in investigating whether there is any correlation between these two proteins that both have a positive effect on tumor growth. In this study, we illustrated that ZBTB7A positively regulates the expression of CD95 and the growth of colon cancer cells, providing a potential target for the treatment of colon cancer.

## Materials and methods

### Plasmids and reagents

For expression of the ZBTB7A protein, full-length human ZBTB7A cDNA fragment was PCR-amplified from a cDNA library of HT-29 and cloned into the pLvx-IRES-puro- Vector (our lab stock).

Reagents were obtained from the following sources: Curcumin (53828ES60, Yeasen), anti-β-actin antibody (5125S, CST); anti-ZBTB7A (ab175918, Abcam), anti-GAPDH (sc-32233,Santa cruz), anti-CD95,(8023S, CST) anti-JNK2 (9258S, CST); anti-Caspase2 (bs-5802R, Bioss), anti-NF-κB-P65(sc-372, Santa cruz), anti-p-NF-κB-P65(3033S,CST)

### Cell culture

Human colorectal carcinoma cell line HCT116, HT29, HCT8, SW620 were cultured in Dulbecco’s modiﬁed Eagle’s medium (DMEM) (Gbico) supplemented with 10% fetal bovine serum (FBS) (Bioin) at 37°C in a 5% CO2 incubator.

### Generation of ZBTB7A knockdown Cell lines

ZBTB7A knockdown cell lines were established by using a lentivirus-based CRISPR/Cas9 system. The single guide RNA (sgRNA) sequences:

Primer-pair2: 5’-GACCAGGGCGCCGCTGAAGTACTTCGGGCTGCAA-3’ (forward) and 5’-TTGCAGCCCGAAGTACTTCAGCGGCGCCCTGGTC-3’ (reverse).
Primer-pair3: 5’-GACCAACAAGCTGAAGGTGCACATGGGGCTGCAA-3’ (forward) and 5’-TTGCAGCCCCATGTGCACCTTCAGCTTGTTGGTC-3’ (reverse).

Annealed double-stranded sgRNA oligonucleotides were ligated into the lentiCRISPRv2 vector (Addgene plasmid 52961), which co-expresses Cas9 and sgRNA in the same vector. Subsequently, HCT116 cells were infected with sgRNA-encoding lentivirus and cultured in DMEM supplemented with 10% FBS. The infected cells were cultured in DMEM containing 1 mg/mL puromycin for 14 days of selection. Surviving cells were selected and expanded for sequencing analysis.

### Growth curve assay

The cell proliferation rates were measured by using cell counting kit 8 (Beyotime) to generate cell growth curves. In brief, cells were seeded into 96-well plates at a density of 10000 cells/well in 100 uL culture medium. Cell proliferation was determined at 0, 24, 48, and 72 h according to the manufacturer’s protocol. The absorbance at 450 nm was measured using a microplate reader (Bio-Tek). Quantitation was obtained from three biological replicates.

### Transfection

700,000 cells were seeded in 12 well plates one day prior to transfection. Cells were transfected with 1 μg of plasmid using PEI (Polysciences). After 48 h post-transfection, cells were harvested for subsequent analysis.

### Annexin V Staining

The Annexin V-APC/propidium iodide (PI) kit (invitrogen) was used to determine apoptotic cell death. The HCT116 cell line and ZBTB7A-konckdown cell lines were collected and resuspended in 1x Binding Buffer at 5x10^6^ cells/mL, 5 μL of Annexin V was subsequently added into the samples, followed by incubation for 15 min at room temperature in the dark. After the cells were washed with 1x Binding Buffer and added with 5 μL of PI, cells were assessed using a flow cytometer (Celesta, BD Biosciences).

### Western blotting

Cells were washed with PBS and then lysed in 1 x SDS loading buffer. Equal amounts of proteins were separated by SDS-PAGE and transferred to a polyvinylidene diﬂuoride (PVDF) membrane. Membranes were incubated with the specific antibodies overnight at 4°C, followed by the detection with secondary antibodies conjugated to horseradish peroxidase (HRP). Proteins were visualized using enhanced chemiluminescence (ECL) detection agents (Millipore).

### ChIP assay

1 × 10^7^/ml cells were fixed in 1% formaldehyde (Invitrogen, USA) for 10 min at room temperature, and 1/10 volume of 1.3 M glycine (Sangon Biotech) was added for 10 min to quit formaldehyde. Then the samples were washed by PBS,and incubated for 30 min in ChIP lysis buffer (50 mM Tris-HCl pH 8.0, 5 mM EDTA, 150 mM NaCl, 1% NP-40, 0.1% SDS, protease inhibitor) on ice, the lysates were sheared by sonication using a Bioruptor (Vibra cell). Cross-linked chromatin samples were incubated with A/G-conjugated agarose beads (invitrogen) or anti-Flag M2 beads (sigma) overnight in the rotator at 4°C, then collecting the beads and washing three times. To elute DNA fragments, immunocomplexes were incubated with elution buffer (50 mM Tris-HCl, pH 8.0, 10 mM EDTA, 1.0% SDS) for 2 h at 65°C, one of the eluted immunocomplexes was saved as immunoprecipitation sample, the other was treated with proteinase K (Sigma-Aldrich) 5h at 55°C. Finally, the DNA was purified with a TIANamp Genomic DNA Kit (TIANGEN Biotech). The purified DNA was detected by qPCR.

### Real-time qPCR

Total RNA was extracted using Trizol reagent (TIANGEN) and 1 µg of total RNA was used to generate cDNA using the High-Capacity cDNA Reverse Transcription Kit (Toyobo). The mRNA expression levels were quantified utilizing the TaqMan Gene Expression Assay kit (Toyobo) for each gene on a 7500 quantitative Real-time qPCR Machine and SDS software (Applied Biosystems). The mRNA levels were normalized to GAPDH using the manufacturer’s software (Applied Biosystems).

### Statistical analysis

Statistical analysis was performed in Prism (Graph Pad Software). The data was reported as means and standard deviations (SD). Differences between groups of research subjects were analyzed for statistical significance with two-tailed Student t tests or ANOVA tests. A *P* value of 0.05 was considered significant.

## Result

**Fig 1:**
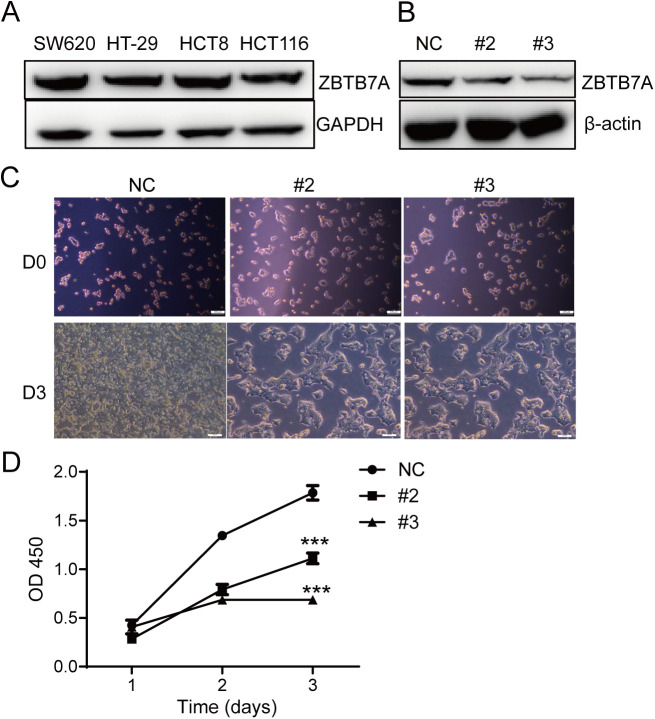
ZBTB7A-knock down inhibits HCT116 cell growth. (A) Western blot analysis of expression level of ZBTB7A in colon cell lines. (B) Western blot analysis of HCT116 ZBTB7A knock-down cell line. (C) Phase-contrast image was performed 3 days after cell culture. Three independent cultures were performed, and representative results are shown. (D) CCK8 assays of HCT116 or ZBTB7A-knockdown HCT116 cell lines 1,2,3 days after cell culture. *p* value was determined by with ANOVA tests OD, optical density. Two independent experiments were quantiﬁed, yielding similar results.

**Fig 2:**
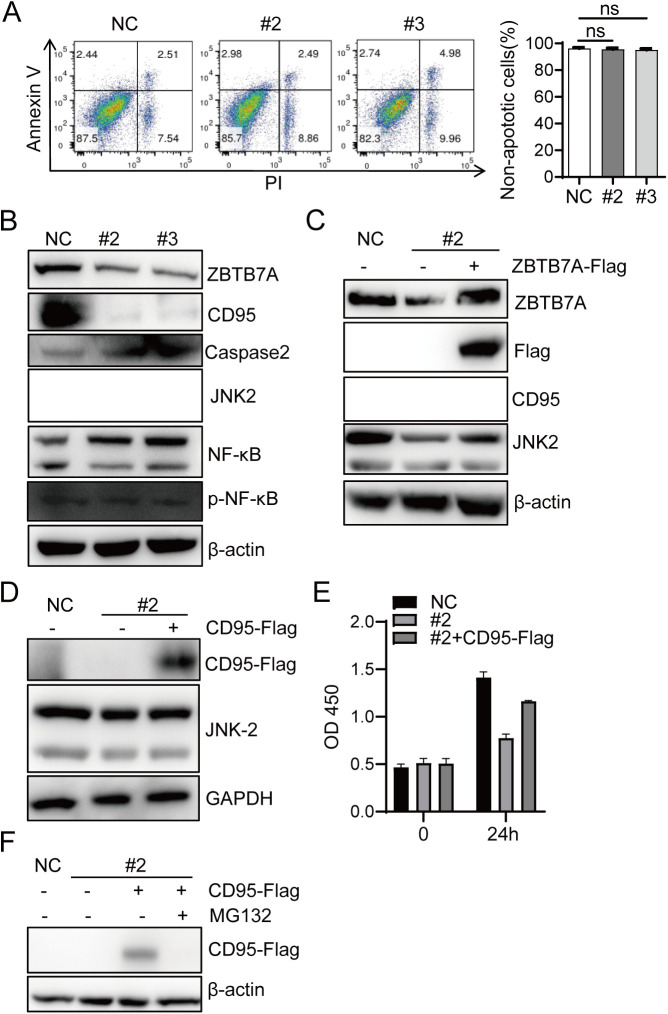
ZBTB7A regulates CD95-mediated cell growth signaling. (A) Cell apoptosis was detected using Annexin V Staining.Left:Representative dotplots of annexin V and PI staining for HCT116-NC and HCT116-ZBTB7A KD cells 3 days after cell culture. Right: Quantiﬁcation of live HCT116 cells. HCT116-NC and HCT116-ZBTB7A KD cells were cultured for 3 days, harvested subjected to apoptosis analysis by annexin V and PI staining. The histogram represents means and SD of three independent cultures (three experiments) *p* value was determined by with ANOVA tests (B) Western blot analysis of CD95, capsase2, JNK2, and β-actin in HCT116 parental and HCT116 ZBTB7A knockdown cell lines 3 days after cell culture. C Western blot analysis of CD95, JNK2, and β-actin in HCT116 parental and HCT116 ZBTB7A knockdown cell #2 2 days after transfection with the pLVX or pLVX-ZBTB7A-Flag.FLAG tag: an 8-amino acid peptide epitope, is genetically fused to target proteins. (D) Western blot analysis of CD95-Flag, JNK2 and β-actin in HCT116 parental and HCT116 ZBTB7A knockdown cell lines2 days after transfection with the pLVX or pLVX-CD95-Flag. (E) CCK8 assays of HCT116 or ZBTB7A-knockdown HCT116 #2 cell lines 0.24h after transfection with pLVX or pLVX-CD95-Flag. (F) Western blot analysis of CD95-Flag and β-actin in HCT116 parental and HCT116 ZBTB7A knockdown cell lines#2. 6h post transfected with pLVX or pLVX-CD95-Flag, DMSO or MG132 was added in the cells, then the cells were culture for 2 days, harvested subjected to analysis.

To investigate the role of ZBTB7A in colon cancer cells, we firstly detected its expression in SW620, HT-29, HCT8, and HCT116 colon cancer cells. Immunoblot analysis confirmed ZBTB7A expression inall tested cell lines (Fig 1A). To further explore its function, we used the CRISPR-Cas9 gene editing system to establish two ZBTB7A knockdown HCT116 cell lines, #2 and #3 (Fig 1B). The growth rate of ZBTB7A knockdown cell lines was significantly reduced compared to the control group. (Fig 1C and 1D). To determine whether this growth inhibition involved cell death, we performed Annexin V/PI staining assays. Notably, both knockdown cell lines and controls showed similar Annexin V^+^PI^+^ populations, approximately 2–5%, suggesting that ZBTB7A knockdown did not induce apoptosis (Fig 2A).

Previous study has reported that CD95 deficiency in some cell lines such as HCT116 can trigger mitochondrial ROS, activate the Caspase2 pathway, and induce cell necrosis [9]. CD95-mediated non-apoptotic signaling has been shown to involve involve NF-κB activation and stimulation of three MAP kinase pathways, Erk1/2, JNK1/2, and p38 [10], To investigate whether ZBTB7A knockdown affects CD95 expression, we performed Western blot analysis. Intriguingly, ZBTB7A depletion led to a marked reduction in CD95 protein levels, along with a modest increase in Caspase2 expression and decreased JNK2 levels (Fig 2B). While we observed elevated levels of potential NF-κB modification forms in ZBTB7A-knockdown cells, the activated form of NF-κB showed no significant increase (Fig 2B). Notably, ZBTB7A overexpression partially rescued both CD95 and JNK2 expression in knockdown cells (Fig 2C). Similarly, CD95 overexpression alone was sufficient to partially restore JNK2 levels (Fig 2D) and promote cell growth (Fig 2E). Furthermore, proteasome inhibition by MG132 treatment failed to increase CD95 expression in ZBTB7A-knockdown cells, with CD95 becoming undetectable (Fig 2F). This finding strongly suggests that ZBTB7A regulates CD95 at the transcriptional level rather than through post-translational modification.

**Fig 3:**
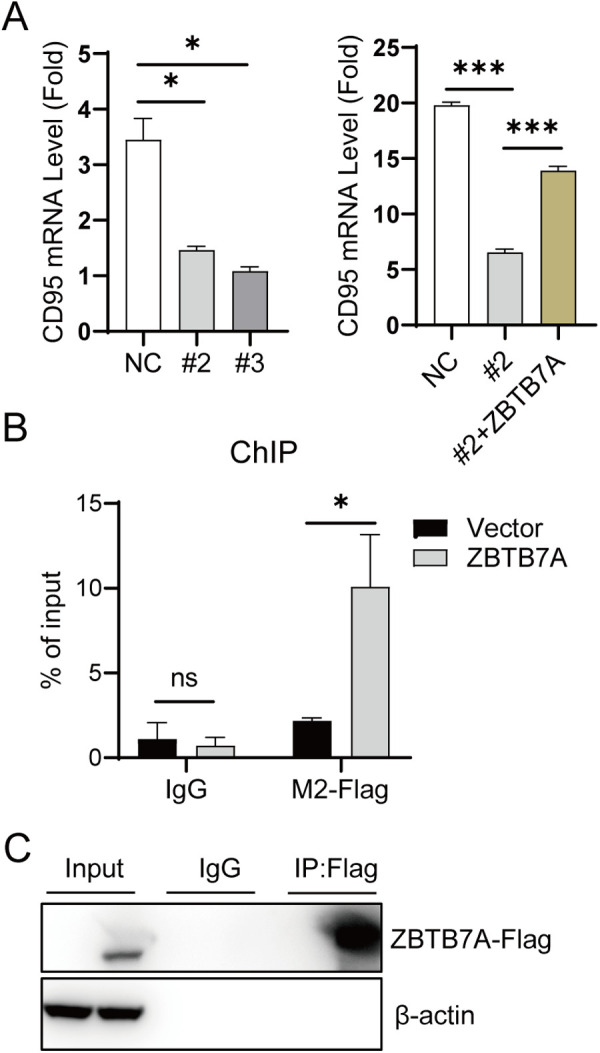
ZBTB7A activates CD95 transcription in HCT116 cell line. (A) The mRNA level of CD 95 in HCT116 cells (ZBTB7A knockdown) was quantified using real time PCR. CD95 mRNA level in HCT116 parental and HCT116-ZBTB7A knockdown cell #2, along with or without ZBTB7A-Flag expression 2 days after transfection with the pLVX or pLVX-ZBTB7A-Flag. *p* value was determined by with ANOVA tests (B) HCT116 cells were transfected with pLVX or pLVX-ZBTB7A-Flag, then the cells were harvested at 2 days post transfection and analyzed by ChIP assay with anti-Flag and anti-rabbit IgG, respectively. And the ChIP qPCR results were shown as % of input. *p* value was determined by with two-tailed unpaired Student’s t-test. (C) Immunocomplexes were subjected to immunoprecipitation using anti-Flag antibody and detected by western blot. *p < 0.05, **p < 0.01 and ***p < 0.001. ChIP, chromatin immunoprecipitation.

To further determine how ZBTB7A regulates the expression of CD95, RT-qPCR analyses were performed to detect mRNA expression of CD95. Strikingly, ZBTB7A knockdown resulted in significant downregulation of CD95 mRNA (Fig 3A). (Fig 3A). Furthermore, ZBTB7A overexpression could rescue the mRNA expression of CD95, demonstrating transcriptional regulation of CD95 by ZBTB7A (Fig 3A). Additionally, as shown in Fig 3B and 3C, a ChIP assay confirmed that ZBTB7A was recruited to the CD95 promoter region (791–1039). These data indicate that ZBTB7A directly binds to the promoter region of CD95 and regulates its expression.

**Fig 4:**
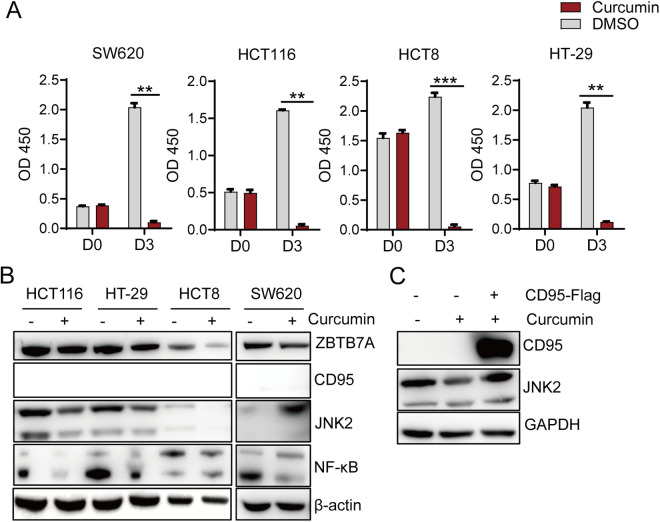
ZBTB7A inhibitor curcumin can induce colorectal cells death. (A) Cell proliferation was assessed by a CCK8 assay in several colorectal cell lines at the indicated times. *p* value was determined by with two-tailed unpaired Student’s t-test. (B) Western blot analysis for ZBTB7A, CD95, JNK2, NF-κB, and β-actin of several colorectal cell lines that they were stimulated with curcumin(50uM) for 24h. (C) Western blot analysis of CD95-Flag, JNK2. and β- actin in HCT116 cell lines.24h after transfection with the pLVX or pLVX-ZBTB7A-Flag, the cells was treated with curcumin (50uM) for another 24h and harvested subjected to analysis.

Curcumin has been identified as a potential ZBTB7A inhibitor [11]. To further verify the function of ZBTB7A, we treated four colon cancer cell lines, HCT8, HCT116, HT-29, and SW620 with curcumin. Notably, curcumin treatment significantly suppressed proliferation in all cell lines tested (Fig 4A). Consistent with ZBTB7A’s regulatory role, CD95 and JNK2 expression were concomitantly downregulated in CD95-expressing cell lines, HCT8, HCT116, and HT-29, following curcumin treatment (Fig 4B). Importantly, CD95 overexpression partially rescued curcumin-mediated JNK2 suppression in HCT116 cells (Fig 4C). These findings collectively indicate that pharmacological inhibition of ZBTB7A effectively suppresses colon cancer cell growth.

## Discussion

Our study demonstrates that ZBTB7A depletion reduces CD95 expression and suppresses colorectal cancer cell growth In HCT116 cells, ZBTB7A knockdown concurrently decreased JNK2 levels (Fig 2B), suggesting its additional role in modulating cell survival/death through JNK2 signaling. Interestingly, while SW620 cells exhibit minimal CD95 expression, they remain sensitive to curcumin-induced cell death (Fig 4A and 4B), implying that NF-κB-mediated survival signaling may function independently of the ZBTB7A-CD95 axis through alternative pathways. It may indicated that the survival signaling of NF-κB in colorectal cells appears to operate independently of ZBTB7A and CD95, suggesting alternative pathways may be involved in cell survival mechanisms. The dual role of JNK2 in tumor biology is well-documented-it can either promote proliferation or induce apoptosis depending on cellular context [12].We speculate that in CD95-expressing colorectal cancer cells, JNK2 primarily sustains proliferative signaling, while in CD95-deficient cells, JNK2 preferentially activates cell death pathways. This hypothesis is supported by our observation that ZBTB7A-knockdown HCT116 cells show markedly reduced exogenous CD95 expression compared to wild-type counterparts (Figs 2F and 4C), further confirming ZBTB7A’s direct regulation of CD95 (Fig 4B). Several important questions remain for future investigation:

The precise mechanism by which curcumin inhibits ZBTB7A activity and induces cell death requires elucidation. In vivo validation using animal models will be essential to confirm the ZBTB7A-CD95 axis’s therapeutic potential. IN summary, our findings establish ZBTB7A as a promising molecular target for colorectal cancer therapy, acting through its regulation of both CD95 and JNK2 signaling pathways.

## Supporting information

S1 File Orginal western blot data of Figs 1, 2, 3 and 4. (ZIP)

## References

[1] Siegel RL, Miller KD, Fuchs HE, Jemal A. Cancer statistics, 2022. CA Cancer J Clin. 2022;72:7–33. 35020204 10.3322/caac.21708

[2] Hou W, Yi C, Zhu H. Predictive biomarkers of colon cancer immunotherapy: present and future. Front Immunol. 2022;13:1032314. doi: 10.3389/fimmu.2022.1032314 36483562 PMC9722772

[3] Singh AK, Verma S, Kushwaha PP, Prajapati KS, Shuaib M, Kumar S, et al. Role of ZBTB7A zinc finger in tumorigenesis and metastasis. Mol Biol Rep. 2021;48(5):4703–19. doi: 10.1007/s11033-021-06405-x 34014468

[4] Zhu M, Wang P, Feng F, Li M-Y. LRF inhibits p53 expression in colon cancer cells via modulating DAP5 activity. Cell Biochem Funct. 2017;35(7):401–6. doi: 10.1002/cbf.3287 28849590

[5] Liebl MC, Hofmann TG. The role of p53 signaling in colorectal cancer. Cancers (Basel). 2021;13. 10.3390/cancers13092125 PMC8125348 33924934

[6] Strasser A, Jost PJ, Nagata S. The many roles of FAS receptor signaling in the immune system. Immunity. 2009;30(2):180–92. doi: 10.1016/j.immuni.2009.01.001 19239902 PMC2956119

[7] Chen L, Park S-M, Tumanov AV, Hau A, Sawada K, Feig C, et al. CD95 promotes tumour growth. Nature. 2010;465(7297):492–6. doi: 10.1038/nature09075 20505730 PMC2879093

[8] Hoogwater FJH, Steller EJA, Westendorp BF, Borel Rinkes IHM, Kranenburg O. CD95 signaling in colorectal cancer. Biochim Biophys Acta. 2012;1826(1):189–98. doi: 10.1016/j.bbcan.2012.03.007 22498253

[9] Hadji A, Ceppi P, Murmann AE, Brockway S, Pattanayak A, Bhinder B, et al. Death induced by CD95 or CD95 ligand elimination. Cell Reports. 2014;7:208–22. 24656822 10.1016/j.celrep.2014.02.035 PMC4083055

[10] Peter ME, Hadji A, Murmann AE, Brockway S, Putzbach W, Pattanayak A, et al. The role of CD95 and CD95 ligand in cancer. Cell Death Differ. 2015;22(4):549–59. doi: 10.1038/cdd.2015.3 25656654 PMC4356349

[11] Cui J, Meng X, Gao X, Tan G. Curcumin decreases the expression of Pokemon by suppressing the binding activity of the Sp1 protein in human lung cancer cells. Mol Biol Rep. 2010;37(3):1627–32. doi: 10.1007/s11033-009-9575-6 19444642

[12] Bubici C, Papa S. JNK signalling in cancer: in need of new, smarter therapeutic targets. Br J Pharmacol. 2014;171(1):24–37. doi: 10.1111/bph.12432 24117156 PMC3874694

